# Autologous hematopoietic cell transplantation for relapsed multiple myeloma performed with cells procured after previous transplantation–study on behalf of CMWP of the EBMT

**DOI:** 10.1038/s41409-022-01592-y

**Published:** 2022-02-15

**Authors:** Joanna Drozd-Sokołowska, Luuk Gras, Nienke Zinger, John A. Snowden, Mutlu Arat, Grzegorz Basak, Anastasia Pouli, Charles Crawley, Keith M. O. Wilson, Herve Tilly, Jennifer Byrne, Claude Eric Bulabois, Jakob Passweg, Zubeyde Nur Ozkurt, Wilfried Schroyens, Bruno Lioure, Mercedes Colorado Araujo, Xavier Poiré, Gwendolyn Van Gorkom, Gunhan Gurman, Liesbeth C. de Wreede, Patrick J. Hayden, Meral Beksac, Stefan O. Schönland, Ibrahim Yakoub-Agha

**Affiliations:** 1 Central Clinical Hospital, The Medical University of Warsaw, Warsaw, Poland; 2 EBMT Statistical Unit Data Office, Leiden, the Netherlands; 3 EBMT Data Office, Leiden, the Netherlands; 4 Department of Haematology, Sheffield Teaching Hospitals NHS Trust, Sheffield, UK; 5 Florence Nightingale Sisli Hospital, Istanbul, Turkey; 6 Haematology Department, “St Savvas” Oncology Hospital, Athens, Greece; 7 Addenbrookes Hospital, Cambridge, UK; 8 Department of Haematology, Cardiff, UK; 9 Centre Henri Becquerel, Rouen, France; 10 Nottingham University, Nottingham, UK; 11 CHU Grenoble Alpes – Université Grenoble Alpes, Grenoble, France; 12 University Hospital, Basel, Switzerland; 13 Gazi University Faculty of Medicine, Ankara, Turkey; 14 Antwerp University Hospital (UZA), Antwerp_Edegem, Belgium; 15 Techniciens d’Etude Clinique suivi de patients greffes, Strasbourg, France; 16 Hospital U. Marqués de Valdecilla, Santander, Spain; 17 Cliniques Universitaires St. Luc, Brussels, Belgium; 18 University Hospital Maastricht, Maastricht, the Netherlands; 19 Ankara University Faculty of Medicine, Ankara, Turkey; 20 Department of Biomedical Data Sciences, Leiden University Medical Center, Leiden, the Netherlands; 21 Department of Haematology, Trinity College Dublin, St. James’s Hospital, Dublin, Ireland; 22 Medizinische Klinik u. Poliklinik V, University of Heidelberg, Heidelberg, Germany; 23 CHU de Lille, Univ Lille, INSERM U1286, Infinite, 59000 Lille, Lille, France

**Keywords:** Stem-cell research, Myeloma

## Abstract

Autologous hematopoietic cell transplantation (auto-HCT) may be performed in multiple myeloma (MM) patients relapsing after a previous auto-HCT. For those without an adequate dose of stored stem cells, remobilization is necessary. This retrospective study included patients who, following disease relapse after the first auto-HCT(s), underwent stem cell remobilization and auto-HCT performed using these cells. There were 305 patients, 68% male, median age at salvage auto-HCT was 59 years. The median time to relapse after the first-line penultimate auto-HCT(s) was 30.6 months, the median follow-up after salvage auto-HCT 31 months. The 2- and 4-year non-relapse mortality (NRM) after the salvage auto-HCT was 5 and 9%, the relapse incidence 56 and 76%, respectively. Overall survival (OS) after 2 and 4 years was 76 and 52%, progression-free survival (PFS) 39 and 15%. In multivariable analysis an increasing interval between the penultimate auto-HCT and relapse was associated with better OS and PFS, later calendar year of salvage auto-HCT with better OS. In conclusion, salvage auto-HCT performed with cells remobilized after a previous auto-HCT was associated with acceptable NRM. The leading cause of failure was disease progression of MM, which correlated with a shorter interval from the penultimate auto-HCT to the first relapse.

## Introduction

Autologous hematopoietic cell transplantation (auto-HCT) is the standard of care in the treatment of eligible patients with multiple myeloma. It is usually incorporated into the first-line treatment, either as a single or a tandem auto-HCT [1, 2]. Despite the availability of many novel therapies, auto-HCT retains a role in patients relapsing after a previous auto-HCT, assuming the relapse-free interval after the first auto-HCT(s) was sufficiently long and lasted at least 18 months if not on any treatment, or at least 36 months, if the patient was on maintenance lenalidomide [1].

For patients considered suitable for a salvage auto-HCT, there may, however, be either an insufficient remaining stem cell dose or none at all in storage. Remobilization to procure new cells is then required. However, data on the efficacy of remobilization are scarce [3–8]. Although some reports analysing the efficacy of salvage auto-HCT included patients in whom hematopoietic cells were harvested during remobilization performed after a prior auto-HCT [9–15], none of them reported on the specific outcomes of this group of patients.

Research dedicated to this group is extremely scarce [3, 4], as a result of which there is limited data on the efficacy and safety of salvage auto-HCT performed with remobilized stem cells. While there is no clinical evidence to suggest that the efficacy of auto-HCT performed with cells procured after previous high-dose therapy is different from auto-HCT performed with cells collected prior to the first transplantation, safety remains a concern. In the short term, is engraftment delayed? In the longer term, is there an increased rate of second primary malignancies (SPM), especially therapy-related myeloid neoplasms i.e., myelodysplastic syndromes (t-MDS) or acute myeloid leukemia (t-AML)? We, therefore, studied efficacy and safety after salvage auto-HCT in a retrospective cohort of multiple myeloma patients.

## Materials and methods

### Data source

The study was performed on behalf of the Chronic Malignancies Working Party (CMWP) of the EBMT. EBMT is a voluntary organization comprising more than 500 transplant centres from Europe and beyond. Accreditation as a member center requires submission of minimal essential data (MED-A form) from all consecutive patients to a central database.

Member centers were invited to provide additional study-specific data about eligible patients, specifically information about stem cell collection, conditioning, and remobilization of stem cells. EBMT Centres commit to obtain informed consent according to the local regulations applicable at the time in order to report pseudonimysed data to the EBMT.

### Study Population and outcome

This study was a retrospective analysis of all myeloma patients who had had an auto-HCT (single or tandem) and who, following disease progression, went on to undergo stem cell remobilization and an auto-HCT performed with cells procured during the remobilization (“new cells”). Patients who received a mixture of new cells and cells mobilized before the first auto-HCT and stored afterward were also eligible. Only patients whose interval between the penultimate auto-HCT and the subsequent remobilization was longer than six months were considered eligible. The analysis includes salvage auto-HCTs performed between 2000 and 2018.

The first auto-HCT was defined as the initial auto-HCT performed for patients first-line, while the second auto-HCT as the last of the two tandem auto-HCTs performed in frontline. The penultimate auto-HCT was the last auto-HCT performed prior to the salvage auto-HCT, meaning that it could be either the first auto-HCT or the second auto-HCT, depending on the clinical situation.

The primary objective of the study was to assess non-relapse mortality (NRM). The secondary objectives were to examine timing of recovery, overall survival (OS), relapse incidence (RI), progression free survival (PFS), the cumulative incidence of t-MDS and t-AML (t-MDS/t-AML CI), and the cumulative incidence of other secondary primary malignancies (SPM CI).

### Statistical analysis

All time-to-event outcomes were computed from the day of the salvage auto-HCT given after remobilization. OS was defined as the time from salvage auto-HCT to death from any cause and PFS was defined as the time from auto-HCT to relapse or progressive disease or death from any cause, whichever came first. Time to t-MDS/t-AML was defined as the time from salvage auto-HCT to therapy-related myelodysplastic syndrome or acute myeloid leukemia. Time to SPM was similarly defined, whilst time to any secondary malignancy was defined as the time to the first occurrence of either t-MDS/t-AML or SPM after salvage auto-HCT.

The Kaplan–Meier estimator and log-rank test were used for OS and PFS, and the crude cumulative incidence estimator and Gray’s test were used for competing events (progression/relapse and NRM; t-MDS/t-AML incidence and death without t-MDS/t-AML; SPM incidence and death without SPM; any secondary malignancy cumulative incidence, and death without any secondary malignancy). The median follow-up was calculated using the reverse Kaplan–Meier estimator [16].

The quality of data for calculating the cumulative incidence of t-MDS/t-AML and other SPM was checked for patients for whom additional data was obtained (*n* = 130). There was only one extra event discovered in those patients compared to the data already available in the EBMT database. Based on this information, the data quality was found reliable enough to extrapolate and report the results for the whole cohort of patients.

OS and PFS were also analyzed using a multivariable Cox model. Variables considered clinically meaningful and which were significant in univariable analyses were selected for inclusion in the multivariable models.

The unadjusted effect of time between penultimate auto-HCT and relapse on OS and PFS was modeled in two ways: using restricted cubic splines and using a linear effect.

The number of collected CD34 cells was log-transformed to comply with normality assumptions and compared between the first and salvage transplantation using a linear mixed effects (LME) model with a random effect for each patient. This allowed inclusion of patients contributing data to only one of the two transplantations. *P*-values were obtained using Satterthwaites degrees of freedom method.

Timing of recovery was compared between the first, second (if a tandem auto transplantation was performed), and salvage transplantation using Cox proportional hazard frailty models (including a gamma-distributed random effect for each patient) and using the exact method for tied observation times. We also compared timing of recovery after the salvage auto-HCT between patients who were infused with a mixture of old and new CD34 cells and those who were infused with only new CD34 cells using Kaplan–Meier plots and the log-rank test.

*P*-values < 0.05 were considered significant. All estimates are reported with accompanying 95% confidence intervals in brackets. All analyses were performed in R version 3.6.3 [17], using ‘survival’’, ‘cmprsk’’, ‘prodlim’’ and ‘lme4’’ packages.

## Results

### Patients, remobilization

Three hundred and five patients, fulfilling the inclusion criteria, were included in the analysis. Additional data requests allowed for more detailed characterization of a subgroup of 130 patients transplanted in 28 centres, including data on remobilization, collection yield, and the type of cells infused, as described in the section on Data Source in Materials and Methods. Patients with additional data obtained on remobilization and salvage auto-HCT were more likely to be diagnosed and transplanted more recently in comparison to patients for whom no additional data was obtained (Table 1 and Supplementary Table S1).

**Table 1:** Patient characteristics at salvage auto-HCT performed with cells procured after previous auto-HCT(s) (* Missing for patients for whom additional data was not obtained).

	Whole population (*n* = 305)	Patients for whom additional data were obtained (*n* = 130)
Sex		
Male	207 (68%)	85 (65%)
Female	98 (32%)	45 (35%)
Year of salvage auto-HCT		
2000–2004	117 (38%)	18 (14%)
2005–2009	29 (10%)	9 (7%)
2010–2014	85 (28%)	56 (43%)
2015–2018	74 (24%)	47 (36%)
Age at salvage auto-HCT; years, median, IQR	59 (53–63)	59 (54–65)
Age at salvage auto-HCT stratified by the calendar year of salvage auto-HCT; years, median, IQR		
2000–2004	57 (52–62)	54 (50–60)
2005–2009	58 (54–62)	57 (51–63)
2010–2014	60 (56–65)	60 (56–67)
2015–2018	60 (53–66)	61 (53–66)
Number of lines of therapy between previous auto-HCT and salvage auto-HCT		
1	132 (49%)	67 (53%)
2	86 (32%)	47 (37%)
3	35 (13%)	10 (8%)
4	7 (3%)	1 (1%)
≥5	7 (3%)	1 (1%)
Number of lines of therapy between previous auto-HCT and salvage auto-HCT; median, IQR	2 (1–2)	1 (1–2)
Radiotherapy any time before salvage auto-HCT	74 (32%)	40 (41%)
Conditioning		
Melphalan only	292 (96%)	126 (97%)
Melphalan in combination	12 (4%)	4 (3%)
No melphalan	1 (0%)	0 (0%)
Stem cell source		
Peripheral blood	293 (97%)	127 (99%)
Bone marrow	4 (1%)	0 (0%)
Peripheral blood + bone marrow	6 (2%)	1 (1%)
Total infused CD34+, x10^6^/kg		
Median, range	2.92 (1.07–24.5)	2.74 (1.27–9.42)
Missing	52 (17%)	19 (15%)
Type of cells infused	*	
Only new cells	96 (86%)	96 (86%)
Mixture of old and new cells	15 (14%)	15 (14%)

There were 207 (68%) males and the median age at salvage auto-HCT was 59 (range 32–78) years. Median age at salvage auto-HCT increased with later calendar years (Table 1). Patients’ characteristics at diagnosis of multiple myeloma are presented in Supplementary Table S1. Before salvage remobilization and auto-HCT 259 (85%) patients had been treated with single and 46 (15%) with tandem auto-HCT. The median time to relapse after the penultimate auto-HCT was 30.6 (1.2–147.3) months, whereas the median time between the penultimate auto-HCT and salvage remobilization was 43.9 (range 7.1–152) months. There were 19 patients remobilized within < 18 months, including 4 patients remobilized within eight months. Fifty-nine (19%) patients were either in a complete remission (CR) or a very good partial remission (VGPR) at remobilization, while a further 154 (50%) were in partial remission (PR). Importantly, data on VGPR was only collected for patients transplanted in 2010 or later. Two hundred and seventy-five (90%) patients underwent a single remobilization, 28 (9%) two and only two (1%) patients had three or more remobilization attempts. While the first remobilization was usually performed with chemotherapy (54%), the second mobilization was performed with G-CSF and/or plerixafor in 79%. Plerixafor was used in 52 (17%) patients in any remobilization attempt. The median total collection was 3.39 (range 0.24–16.0) x 10^6^ CD34 + cells/ kg body weight compared to 4.81 (range 1.63–38.4) x 10^6^ CD34 + cells/ kg body weight obtained before the first auto-HCT (*p* < 0.0001). Further data on remobilization is presented in Supplementary Table S2.

### Salvage auto-HCT

Salvage auto-HCT was performed after single agent high-dose melphalan conditioning in 292 (96%) patients, and melphalan in combination with other drugs in a further 12 (4%) patients. The source of hematopoietic stem cells was the peripheral blood in 293 (96%) patients, and patients received a median dose of 2.92 (1.07–24.5) x 10^6^ CD34+ cells/ kg body weight. Data on the type of stem cells were available for 111 patients, among whom 96 (86.5%) received solely new cells, and 15 (13.5%) both old and new cells. Engraftment failure was noted in seven (2%) patients. All these patients eventually survived beyond 7 months. Five of them experienced a relapse. In the analyzed group, there were also 2 patients who died before engraftment within the first 28 days, and 2 for whom the follow-up was censored at 10 and 11 days before they reached engraftment. These patients were not included in the calculation of the engratment failure ratio.

Time to neutrophil recovery did not differ between the first auto-HCT and salvage auto-HCT (median 12 vs 12 days, HR = 0.93, 95% CI 0.79–1.11, *p* = 0.43), while there was some evidence for a small difference between the second and salvage auto-HCT (median 11 vs 12 days, HR = 1.52, 95% CI 1.02–2.28, *p* = 0.04). Similarly there was no difference in time to platelet recovery > 20 × 10^9^/ L between the first and the salvage transplantation (median 12 vs 13 days, HR = 1.16, 95% CI 0.96–1.40, *p* = 0.14), while there was again a difference between the second and the salvage auto-HCT (median 11 vs 13 days, HR = 2.09, 95% CI 1.36–3.21, *p* < 0.001). A significantly longer time to platelet recovery ≥ 50 × 10^9^/L after the salvage auto-HCT in comparison to the first and second auto-HCT should be interpreted with caution due to frequent missing data resulting probably from the ambulatory follow-up at this stage of patients’ treatment. Data on hematopoietic recovery is presented in Table 2.

**Table 2:** Comparison of collected and infused CD34 + cells and hematopoietic recovery between the first auto-HCT(s) and salvage auto-HCT.

	First auto-HCT	Second auto-HCT (*n* = 46)	Salvage auto-HCT (*n* = 305)	p*	p**
	(*n* = 305)				
**Total collected CD34**+**cells, x10**^**6**^**/kg**					
Median, range	4.81 (1.63–38.4)	not performed	3.39 (0.24–16)	<0.001	NA
Missing	163 (53%)	not performed	175 (57.4%)		
**Total infused CD34** + **cells, x10**^**6**^**/ kg**					
Median, range	4.05 (1.37–43.2)	4.90 (1.93–24.8)	2.92 (1.07–24.5)	<0.001	<0.001
Missing	70 (23%)	16 (35%)	52 (17%)		
**Hematopoietic recovery; median, IQR (days)**					
ANC > 0.5 × 10^9^/L	12 (11–14)	11 (10–13)	12 (11–14)	0.3	0.02
PLT > 20 × 10^9^/L	12 (11–15)	11 (10–12)	13 (11–16)	0.08	<0.001
PLT > 50 × 10^9^/L	16 (13–21)	14 (12–18)	20 (14–34)	<0.001	<0.001

p* - comparison between the first auto-HCT and salvage auto-HCT.

p** - comparison between the second auto-HCT and salvage auto-HCT.

*P*-values for the comparison of collected and infused CD34+ cells between transplantations were obtained using the F-test from an anova on the linear mixed effect model results. Total number of CD34+ cells were log transformed in the models.

*P*-values for the comparison of hematopoietic recovery between transplantations were obtained using chi^2^-tests in Cox proportional hazard frailty models.

NA–not applicable, ANC-absolute neutrophil count, PLT–platelets.

Patients who received a mixture of old and new cells had a shorter time to neutrophil recovery (median 12 vs 13 days, log-rank *p* = 0.01), while time to platelet recovery was unaffected by the type of cells infused (mixture vs only new cells, log-rank *p* = 0.44 for >20 × 10^9^/L, *p* = 0.09 for >50 × 10^9^/L).

The number of infused CD34+ cells did not affect neutrophil recovery (median 12 vs 12 days, *p* = 0.25). Earlier platelet recovery to > 20 × 10^9^/L was associated with higher number of infused CD34+ cells (median 15 vs 12 days, *p* < 0.001 for < 3 vs ≥ 3 × 10^6^ CD34+ cells/kg). Similarly, platelet recovery to > 50 × 10^9^/L was dependent on the number of infused CD34+ cells (median 23 vs 18 days, *p* = 0.01).

### Outcome

**Fig. 1:**
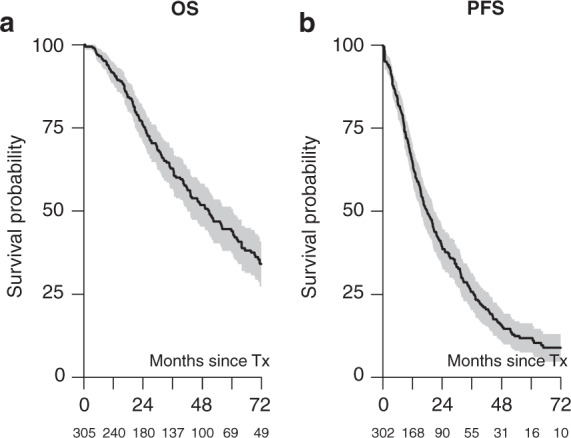
Outcome after salvage auto-HCT performed with remobilized stem cells. **a** Overall survival after salvage auto-HCT performed with remobilized stem cells. **b** Progression-free survival after salvage auto-HCT performed with remobilized stem cells. Numbers below the graph indicate the number of patients at risk.

At a median follow-up of 31 months (95% CI, 25.9–36.4; IQR 13.2–58.4), the 2-, 4- and 6-year non-relapse mortality rates were 5% (95% CI, 2–7%), 9% (95% CI, 5–12%) and 10% (95% CI, 6–14%), respectively. The 100-day and 1-year NRM were 1% (95% CI, 0–2%) and 2% (95% CI, 1–4%), respectively. The cumulative relapse incidence rates at 2, 4 and 6 years were 56% (95% CI, 50–62%), 76% (95% CI, 70–81%) and 81% (95% CI, 76–86%), respectively; see Fig. 1a.

The median OS was 51 months (95% CI, 42–61). OS probabilities were 76% (95% CI, 71–81%), 52% (95% CI, 45–58%) and 34% (95% CI, 27–41%) at 2, 4 and 6 years, respectively. The median PFS was 17 months (95% CI, 15–21). PFS rates at 2, 4 and 6 years were 39% (95% CI, 33–45%), 15% (95% CI, 11–20%) and 9% (95% CI, 5–13%), respectively; see Fig. 1b.

In univariable analysis, the time interval between the penultimate auto-HCT and relapse of greater than 30 months, being the median interval between the penultimate auto-HCT and relapse, was associated with survival benefit in terms of both OS and PFS and a slightly lower relapse incidence. Similarly, the calendar year of salvage auto-HCT impacted both OS and PFS, with patients transplanted before 2005 having inferior outcome. Male gender was associated with lower NRM, but a higher relapse incidence, resulting in no difference in survival. Patients with Durie-Salmon stage III disease at diagnosis had shorter OS. Other factors analyzed were not significantly associated with outcomes in univariable analysis. The detailed results of univariable prognostic factors analysis are presented in Table 3. In multivariable analysis, comprising Durie-Salmon stage at diagnosis (III vs I/II), year of salvage transplantation (per calendar year later) and time interval from the penultimate auto-HCT to relapse (per additional year longer), time to relapse remained significant for both OS and PFS, with respective hazard ratios of 0.84 (95% CI, 0.76–0.94), *p* = 0.002 and 0.90 (95% CI, 0.84–0.98), *p* = 0.009 and year of salvage auto-HCT for OS with hazard ratio equal to 0.95 (95% CI, 0.92–0.99), *p* = 0.005.

**Table 3:** Univariable prognostic factor analysis for overall survival (OS), progression/relapse free survival (PFS), non-relaplse/progression related mortality (NRM) and relapse/progression incidence (RI) with estimates of probabilities/cumulative incidences (95% confidence intervals) at 4 years after salvage auto-HCT.

		OS		PFS		NRM		RI	
			*P*		*P*		*P*		*P*
**Age**			0.17		0.06		0.20		0.55
	<60 years	49% (40–57%)		13% (7–19%)		11% (6–16%)		77% (70–84%)	
	≥60 years	55% (45–65%)		19% (11–27%)		6% (1–11%)		75% (66–83%)	
**Sex**			0.83		0.07		0.005		0.02
	Male	52% (44–60%)		14% (9–20%)		6% (2–10%)		80% (73–86%)	
	Female	51% (40–62%)		18% (9–27%)		14% (6–22%)		68% (57–79%)	
**Year of salvage auto-HCT**			<0.001		0.02		0.20		0.41
	2000–2004	36% (27–46%)		8% (3–14%)		13% (6–19%)		79% (71–87%)	
	2005–2009	61% (42–80%)		14% (1–27%)		4% (0–10%)		82% (68–96%)	
	2010–2014	63% (52–74%)		25% (15–36%)		4% (0–8%)		71% (60–81%)	
	2015–2018	66% (48–83%)		13% (0–27%)		14% (1–27%)		73% (57–90%)	
**Number of previous auto-HCTs**			0.98		0.86		0.94		0.85
	1	52% (45–59%)		16% (11–21%)		9% (5–12%)		76% (69–82%)	
	2	49% (34–65%)		14% (3–24%)		10% (1–19%)		77% (64–90%)	
**Time to relapse after the previous auto-HCT**			<0.001		<0.001		0.24		0.02
	<30 months	35% (26–45%)		10% (3–16%)		12% (6–19%)		78% (70–87%)	
	≥30 months	63% (55–71%)		19% (13–26%)		7% (2–11%)		74% (67–81%)	
**Time interval between the last auto-HCT and salvage auto-HCT**			0.001		0.02		0.03		0.53
	<48 months	41% (33–50%)		12% (7–18%)		13% (7–18%)		75% (68–82%)	
	≥48 months	65% (56–74%)		19% (11–27%)		4% (0–8%)		77% (69–85%)	
**Number of previous lines of therapy**			0.42		0.49		0.22		0.30
	≤3	48% (38–58%)		16% (8–23%)		11% (5–17%)		73% (64–82%)	
	>3	54% (46–62%)		15% (9–21%)		7% (3–12%)		77% (70–84%)	
**Radiotherapy**			0.72		0.65		0.69		0.43
	No	52% (43–62%)		19% (12–26%)		10% (5–16%)		71% (62–79%)	
	Yes	52% (39–65%)		14% (5–23%)		8% (1–15%)		78% (67–88%)	
**Type of infused stem cells**			0.22		0.81		0.55		0.79
	New cells	61% (49–72%)		21% (12–30%)		8% (2–13%)		71% (61–82%)	
	Mixture	79% (59–100%)		18% (0–38%)		0% (0–0%)		82% (62–100%)	
**Number of infused CD34**+ **cells**			0.87		0.61		0.14		0.71
	<3 × 10^6^/kg	53% (43–63%)		18% (10–26%)		6% (1–11%)		75% (67–84%)	
	≥3 × 10^6^/kg	58% (48–69%)		14% (7–21%)		11% (5–18%)		75% (66–84%)	
**Plerixafor in remobilization**			0.57		0.51		0.23		0.16
	No	51% (44–58%)		15% (10–20%)		10% (6–14%)		76% (70–82%)	
	Yes	54% (35–72%)		25% (9–41%)		2% (0–6%)		73% (57–90%)	
**Chemotherapy in remobilization**			0.55		0.42		0.02		0.48
	No	51% (41–60%)		19% (11–27%)		5% (1–9%)		76% (68–84%)	
	Yes	53% (44–62%)		13% (7–19%)		12% (7–18%)		75% (68–83%)	
**Status of MM at remobilization**			0.11		0.30		0.82		0.94
	CR/VGPR/PR*	52% (44–60%)		17% (11–23%)		8% (4–13%)		75% (68–82%)	
	Other	42% (24–59%)		15% (2–27%)		11% (1–22%)		74% (59–89%)	
**Durie–Salmon stage at diagnosis**			0.02		0.16		0.98		0.63
	I or II	59% (49–70%)		18% (10–27%)		8% (2–14%)		73% (64–83%)	
	III	47% (38–56%)		12% (6–18%)		9% (4–14%)		79% (72–86%)	

The 4-year probabilities of OS and PFS were obtained using Kaplan–Meier methods and the 4-year cumulative incidence of NRM and RI was obtained using the crude cumulative incidence estimator. P-values were obtained with the log-rank test for OS and PFS and Gray’s test for NRM and RI and time artificially censored at 6 years.

*VGPR is grouped together with PR and CR vs. Other in order to avoid bias. VGPR has been reported only since 2010.

### Infections and other complications

Infectious complications occurred in 126 patients (50%) among 250 for whom the data was available, with eight patients (6.3%) in this group succumbing to infection. Among 138 reported non-infectious complications, mucositis was the most frequent (35, 25%).

### Secondary malignancies

**Fig. 2:**
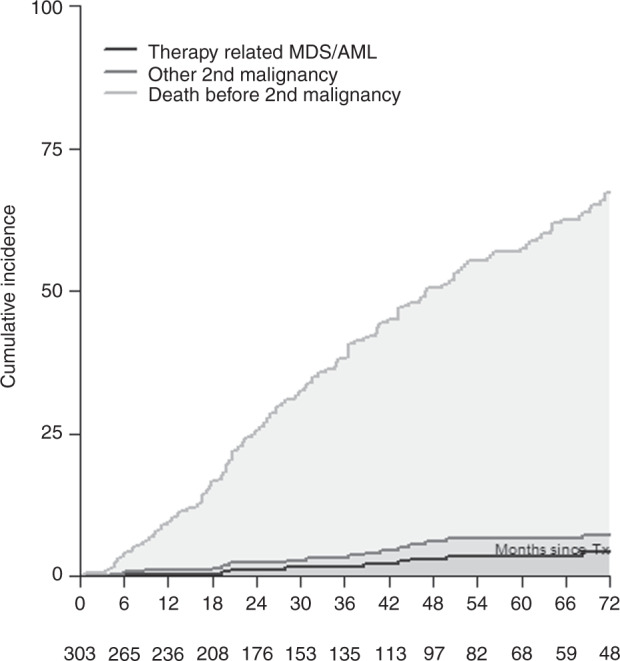
Cumulative incidence of t-MDS/t-AML, non-myeloid second primary malignancy (“other second malignancy”) and death before any second primary malignancy after salvage auto-HCT performed with remobilized stem cells. Numbers below the graph indicate the number of patients at risk.

Twenty out of 303 patients with data on secondary malignancy status available developed secondary malignancies after salvage auto-HCT: five acute leukemias, five myelodysplastic syndromes, one myelodysplastic/ myeloproliferative neoplasm (chronic myelomonocytic leukemia, CMML) and seven solid tumors at a median of 34 months. One patient was diagnosed with a lymphoma, and for the remaining patient, the type of a secondary malignancy was not reported. This translated into a cumulative incidence of t-MDS/t-AML of 1% (95% CI, 0–3%), 3% (95% CI, 1–5%) and 4% (95% CI, 1–7%) at 2, 4 and 6 years, respectively. The cumulative incidence of other SPMs was 1% (95% CI, 0–2%), 3% (95% CI, 1–5%) and 3% (95% CI, 1–5), respectively; see Fig. 2.

There was a trend for patients with Durie Salmon stage III disease at diagnosis to have a higher incidence of t-MDS/t-AML (6% (95% CI 2–11) vs. 0% (95% CI 0–0) at 4 years, 7% (95% CI 2–13) vs. 1% (95% CI 0–4) at 6 years, *p* = 0.055) as Supplementary Table S3 shows. Although the difference was not statistically significant, it is also worth noting that for patients for whom the data on the type of infused cells and secondary malignancy status were available, the 4-year secondary malignancies incidence in the 95 patients who received only new cells was 13%, whilst the incidence in the 15 patients receiving a mixture of old and new cells was 0%.

## Discussion

This retrospective study analyzed the outcomes seen in auto-HCT performed using cells remobilized after a previous hematopoietic cell transplantation. This is the biggest study to date reporting data on this specific patients’ population.

Importantly, all but 2% of the patients treated with this approach engrafted. Time to neutrophil and platelet recovery ≥20 x 10^9^/L did not differ between the first and the salvage auto-HCT. Surprisingly, there was a difference between the second and the salvage auto-HCT, with shorter time to hematopoietic recovery after the second auto-HCT. The reason for this finding is unclear and perhaps results from small patient numbers or from selection bias in those assigned to tandem auto-HCT. As reported previously, [3, 4] we observed a longer time to platelet recovery ≥ 50 × 10^9^/L. This finding should, however, be interpreted with caution due to frequent missing data. Delayed platelet engraftment also was observed by Jimenez-Zepeda [13], who reported a significant difference between platelet engraftment but not neutrophil engraftment between first and salvage auto-HCT. Remarkably, 30 out of 81 patients in their study received remobilized stem cells. Importantly, platelet recovery was influenced by the number of infused CD34+ cells in that patients who received higher CD34+ stem cell doses had more rapid platelet engraftment.

The non-relapse mortality, which was 2%, 5%, 9%, and 10% at 1, 2, 4, and 6 years, respectively, and solely 1% after 100 days, seems acceptable. It is however not possible to compare the NRM obtained in this report with that reported by others, as many studies used both old and new stem cells but reported the outcomes for both types of cells together e.g., [9–15]; alternatively, they only reported on the 100- or even 60-day treatment-related mortality [9, 10, 12–14, 18–21].

The cumulative incidence of t-MDS/t-AML was 1%, 3%, and 4% after 2, 4, and 6 years, respectively (2%, 5%, and 7% for patients for whom additional data was obtained), while for other non-myeloid secondary primary malignancies it was 1%, 3% and 3% respectively. This data indicates that the risk of secondary myeloid malignancies is not excessively high, although not negligible, especially taking into consideration the possibility of underreporting by the centres and intermediate follow-up of our study. Although the cumulative incidence of t-MDS/t-AML was higher than the rate of 1.4% at 6 years reported in the prospective observational CALM (Collaboration to Collect Autologous Transplant outcome in Lymphoma and Myeloma) study by the EBMT [22], it was comparable to the general incidence of t-MDS/t-AML after auto-HCT of 4–5% after 5 years as reviewed in [23]. The incidence of non-myeloid SPMs was below the incidence reported by Sahebi et al [22]. These are important observations given the fact that high dose melphalan was given for the second or even the third time in this patient cohort. It has been postulated, that transient exposure to high doses of melphalan is not deleterious for the patient. This was also inferred from another EBMT study in which a higher dose of melphalan given in conditioning (200 vs 140 mg/m^2^) was not associated with a higher incidence of secondary primary malignancies [24]. It must be also stressed, that when the cumulative incidence of t-MDS/t-AML or other SPMs is compared to the cumulative incidence of death before any secondary malignancy, being 60% after 6 years, it is obvious that t-MDS/t-AML or other SPM are minor causes of treatment failure. This observation was also made by the Center for International Blood and Marrow Transplant Research (CIBMTR) regarding patients receiving salvage auto-HCT, but with stem cells harvested before the first auto-HCT [25]. Therefore, it seems reasonable not to alter any therapeutic decisions in multiple myeloma, including the decision to perform a salvage auto-HCT with remobilized stem cells, based on the risk of secondary primary malignancies. This approach is in line with International Myeloma Working Group (IMWG) consensus guidelines [26].

Interestingly, although the difference did not reach statistical significance, it is worth noting that all secondary malignancies developed in patients who received only new cells. Whether this is a true observation and results from the accumulation of genetic mutations within stem cells in the course of multiple myeloma treatment or is, alternatively, a chance observation merits further study. The calendar year of salvage auto-HCT was significantly associated with the development of secondary malignancies, with patients transplanted before 2005 having the lowest cumulative incidence of both t-MDS/t-AML or any secondary malignancy. This may result from the fact that in this group of patients, the incidence of death before the development of secondary malignancies was almost twice as high as in the later years, reflecting poorer general outcomes following auto-HCT during these earlier years. The increase in median age at auto-HCT over time also may contribute to the increase in the incidence of secondary malignancies.

It is difficult to speculate, how the efficacy of salvage auto-HCT performed with stem cells remobilized after the previous auto-HCT compares with the efficacy of salvage auto-HCT performed with stem cells harvested before the first-line auto-HCT, especially taking into consideration, that for a significant number of studies the information on the type of stem cells used is lacking [21, 27–32]. Nevertheless, median PFS of 17 months and median OS of 51 months in our analysis seem to be within the range reported by others for the salvage auto-HCT [11, 15, 20, 25, 29, 30], including the recent report by CIBMTR with PFS established at 50% at 1 year and 13% at 3 years (vs 65% at 1 year and 26% at 3 years in our study) [32]. Longer time to relapse was associated with survival benefit in terms of both OS and PFS as shown by uni- and multivariable analysis. We also modeled time from penultimate auto-HCT to relapse in a more flexible manner than as a linear variable using restricted cubic splines (results not shown) but found no evidence that the association between time to relapse and OS/PFS was other than linear, indicating that the longer the time to relapse, the better. The phenomenon has also been observed in other studies; for example, in an EBMT study analyzing outcomes after a third auto-HCT performed following previous tandem auto-HCTs, where patients with a longer interval between first-line auto-HCTs and relapse had superior outcomes [21]. Similar correlations have also been seen in other retrospective studies, e.g., [14, 15, 25, 28, 30, 32, 33].

Limitations of the study are its retrospective nature, the possibility of underreporting of the cumulative incidence of t-MDS/t-AML and other SPM, the lack of information on the previous treatment received including novel agents, the lack of cytogenetic data which is likely to impact outcome after salvage-HCT. Because only patients who proceeded to a salvage auto-HCT were selected we do not know how many patients there were with failed remobilization attempts. Additionally, the information on the number of CD34+ cells collected during remobilization is difficult to interpret, because it cannot be excluded, that in some patients with remaining stored stem cells from the first harvest, the collection was stopped earlier at the salvage remobilization. Nevertheless, we believe the study provides important and new information on the safety of salvage auto-HCT performed with cells procured after previous high-dose therapy.

In conclusion, salvage auto-HCT performed with stem cells procured during remobilization after a previous auto-HCT is a viable treatment option when not enough stem cells are cryopreserved. The efficacy is comparable to the efficacy of salvage auto-HCT performed with stem cells harvested before the first auto-HCT and stored afterwards. The non-relapse mortality is acceptable, as are the incidence of secondary malignancies, both t-MDS/t-AML and solid tumors. The leading cause of failure was progression of multiple myeloma, which was associated with shorter time from penultimate auto-HCT to the first relapse.

## Supplementary information

Table S1, Table S2, Table S3
