# Supplementary material for: Autologous hematopoietic cell transplantation for relapsed multiple myeloma performed with cells procured after previous transplantation–study on behalf of CMWP of the EBMT
Source: Bone Marrow Transplant. 2022 Feb 15;57(4):633–40. doi: 10.1038/s41409-022-01592-y (PMC8993690; doi:10.1038/s41409-022-01592-y)
Supplement: Supplementary file 1 — Table S1, Table S2, Table S3 [file 41409_2022_1592_MOESM1_ESM.docx]

**Table S1** Characteristics at diagnosis (A – patients without kidney failure i.e., creatinine concentration ≤2 mg/dL, B – patients with kidney failure i.e., creatinine concentration >2 mg/dL, ISS – International Staging System)

|  | Whole population (n=305) |
| --- | --- |
| Year of diagnosis |  |
| 1985-1999 | 93 (31%) |
| 2000-2004 | 70 (23%) |
| 2005-2009 | 71 (23%) |
| 2010-2015 | 71 (23%) |
| Stage according to Durie-Salmon at diagnosis |  |
| I | 34 (13%) |
| II | 68 (27%) |
| III | 154 (60%) |
| Kidney failure at diagnosis |  |
| A | 225 (88%) |
| B | 31 (12%) |
| ISS at diagnosis |  |
| I | 43 (43%) |
| II | 32 (32%) |
| III | 24 (24%) |

**Table S2** Characteristics of salvage remobilization and transplantation (ANC – absolute neutrophil count, CR – complete remission, G-CSF – granulocyte colony stimulating factor, NR – not reported, PD – progressive disease, PR – partial remission, SD – stable disease, VGPR – very good partial remission)

|  | Whole population |
| --- | --- |
|  | (n=305) |
| Age at remobilization; years, median (range) | 58 (32-78) |
| Number of previous auto-HCT |  |
| 1 | 259 (85%) |
| 2 | 46 (15%) |
| Year of the first autologous transplant |  |
| 1985-1999 | 78 (26%) |
| 2000-2004 | 72 (24%) |
| 2005-2009 | 69 (23%) |
| 2010-2015 | 86 (28%) |
| Time from diagnosis to first transplant; median (range); months | 7.0 (1.6-166) |
| Conditioning for the first transplantation |  |
| Melphalan only | 288 (96%) |
| Melphalan-based | 12 (4%) |
| Conditioning for the second non-salvage transplantation |  |
| Melphalan only | 41 (93%) |
| Melphalan in combination | 2 (5%) |
| Melphalan-free protocol | 1 (2%) |
| Median time between the last non-salvage auto-HCT and first progression, months (range) | 30.6 (1.2-147.3) |
| Total dose of melphalan received before remobilization, mg/m2 |  |
| 100 | 6 (2%) |
| 140 | 41 (16%) |
| 200 | 175 (70%) |
| >200 | 29 (12%) |
| Time between penultimate auto-HCT and 1^st^ remobilization; months, median (range) | 43.9 (7.1-152) |
| Status of multiple myeloma at remobilization |  |
| CR | 17 (7%) |
| VGPR | 42 (16%) |
| PR | 154 (60%) |
| SD/PD | 45 (17%) |
| Peripheral blood parameters at first remobilization; median (range) |  |
| Hemoglobin; g/dL | 12 (7.1-18.8) |
| Platelets; x 10^9^/L | NR |
| ANC; x 10^9^/L | NR |
| Number of remobilization attempts |  |
| 1 | 275 (90.2%) |
| 2 | 28 (9.2%) |
| 3 | 1 (0.3%) |
| 4 | 1 (0.3%) |
| 1^st^ Remobilization |  |
| G-CSF monotherapy | 107 (35%) |
| Plerixafor alone | 2 (1%) |
| G-CSF+Plerixafor | 30 (10%) |
| Chemotherapy + G-CSF | 133 (44%) |
| Chemotherapy + plerixafor | 1 (0%) |
| Chemotherapy+G-CSF+plerixafor | 15 (5%) |
| Chemotherapy alone | 17 (6%) |
| 1^st^ Remobilization* |  |
| Plerixafor | 48 (16%) |
| Chemotherapy-based | 166 (54%) |
| G-CSF monotherapy | 107 (35%) |
| ≥2^nd^ Remobilization |  |
| Total number of remobilizations | 33 |
| G-CSF monotherapy | 13 (39%) |
| Plerixafor alone | 1 (3%) |
| G-CSF+Plerixafor | 10 (30%) |
| Chemotherapy + G-CSF | 6 (18%) |
| Chemotherapy alone | 2 (6%) |
| Chemotherapy+G-CSF+plerixafor | 1 (3%) |
| ≥2^nd^ Remobilization* |  |
| Plerixafor | 12 (36%) |
| Chemotherapy-based | 9 (27%) |
| G-CSF monotherapy | 13 (39%) |
| Patients who received plerixafor in any mobilisation regimen | 52 (17%) |
| Total collected CD34+; Median, range; x 10^6^/ kg | 3.39 (0.24-16) |
| Total collected CD34+, x 10^6^/ kg |  |
| <2 | 15 (11%) |
| 02.maj | 88 (68%) |
| ≥5 | 27 (21%) |
| * Percentages do not add to 100% as the categories are not mutually exclusive. | |

**Table S3** Univariable prognostic factor analysis for t-MDS/t-AML, any secondary malignancy with 4-year cumulative incidence estimates (95% confidence intervals).

|  |  | **t-MDS/t-AML CI** |  | **Any secondary malignancy CI** | |
| --- | --- | --- | --- | --- | --- |
|  |  |  | P |  | P |
| **Age** |  |  | 0.40 |  | 0.25 |
|  | <60 years | 3% (0-6%) |  | 5% (1-9%) |  |
|  | ≥60 years | 3% (0-6%) |  | 7% (2-12%) |  |
| **Sex** |  |  | 0.49 |  | 0.49 |
|  | Male | 3% (0-6%) |  | 6% (3-10%) |  |
|  | Female | 3% (0-6%) |  | 6% (0-11%) |  |
| **Year of salvage auto-HCT** |  |  | 0.10 |  | 0.08 |
|  | 2000-2004 | 1% (0-3%) |  | 3% (0-6%) |  |
|  | 2005-2018 | 8% (0-19%) |  | 8% (0-19%) |  |
| **Number of previous auto-HCTs** |  |  | 0.58 |  | 0.21 |
|  | 1 | 3% (1-6%) |  | 7% (3-11%) |  |
|  | 2 | 2% (0-7%) |  | 2% (0-7%) |  |
| **Time to relapse after the previous auto-HCT** |  |  | 0.60 |  | 0.76 |
|  | <30 months | 2% (0-5%) |  | 5% (1-9%) |  |
|  | ≥30 months | 4% (1-7%) |  | 7% (3-11%) |  |
| **Time interval between the last auto-HCT and salvage auto-HCT** |  |  | 0.43 |  | 0.28 |
|  | <48 months | 2% (0-5%) |  | 4% (1-8%) |  |
|  | ≥48 months | 4% (0-8%) |  | 8% (3-14%) |  |
| **Number of previous lines of therapy** | |  | 0.25 |  | 0.42 |
|  | ≤3 | 1% (0-3%) |  | 4% (0-9%) |  |
|  | >3 | 4% (1-8%) |  | 7% (3-12%) |  |
| **Radiotherapy** |  |  | 0.77 |  | 0.59 |
|  | No | 3% (0-6%) |  | 5% (1-9%) |  |
|  | Yes | 3% (0-8%) |  | 8% (1-15%) |  |
| **Type of infused stem cells** |  |  | 0.55 |  | 0.32 |
|  | New cells | 6% (1-12%) |  | 13% (5-20%) |  |
|  | Mixture | 0% (0-0%) |  | 0% (0-0%) |  |
| **Number of infused CD34+ cells** |  |  | 0.61 |  | 0.36 |
|  | <3 x 10^6/kg | 3% (0-6%) |  | 9% (3-15%) |  |
|  | ≥3 x 10^6/kg | 3% (0-7%) |  | 4% (0-9%) |  |
| **Plerixafor in remobilization** |  |  | 0.94 |  | 0.99 |
|  | No | 3% (1-5%) |  | 6% (3-9%) |  |
|  | Yes | 2% (0-7%) |  | 6% (0-15%) |  |
| **Chemotherapy in remobilization** |  |  | 0.95 |  | 0.08 |
|  | No | 2% (0-4%) |  | 2% (0-4%) |  |
|  | Yes | 4% (1-8%) |  | 10% (5-15%) |  |
| **Status of MM at remobilization** |  |  | 0.46 |  | 0.67 |
|  | CR/VGPR/PR* | 3% (0-6%) |  | 6% (2-10%) |  |
|  | Other | 0% (0-0%) |  | 3% (0-9%) |  |
| **Durie-Salmon stage at diagnosis** | |  | 0.06 |  | 0.15 |
|  | I or II | 0% (0-0%) |  | 5% (0-9%) |  |
|  | III | 6% (2-11%) |  | 9% (4-14%) |  |
| **Incidences were estimated using the obtained using the crude cumulative incidence estimator and competing risks (death without t-MDS/t-AML and death without secondary malignancy, respectively). P-values were obtained with Gray’s test and time artificially censored at 6 years.** | | | | | |
